# The clinical impact of 16S ribosomal RNA PCR and sequencing in the identification of bacterial infections: a 7-year report from a Lebanese tertiary care center

**DOI:** 10.3389/fcimb.2025.1619640

**Published:** 2025-08-07

**Authors:** Nour Youssef, Celina F. Boutros, Fatima Dakroub, Fata Akl, Lina Reslan, Marc Finianos, Mohammad Bahij M. Moumneh, Tarek Bou Dargham, Zeinab El Zein, Amani Haddara, Rawan Korman, Sarah Khafaja, Ghassan Matar, George F. Araj, Ghassan S. Dbaibo

**Affiliations:** ^1^ Center for Infectious Diseases Research (CIDR) and World Health Organization (WHO) Collaborating Center for Reference and Research on Bacterial Pathogens, American University of Beirut, Beirut, Lebanon; ^2^ Department of Pediatrics and Adolescent Medicine, American University of Beirut Medical Center, Beirut, Lebanon; ^3^ Pediatric Infectious Diseases Division, Department of Pediatrics and Adolescent Medicine, American University of Beirut Medical Center, Beirut, Lebanon; ^4^ Department of Experimental Pathology, Immunology, and Microbiology, Faculty of Medicine, American University of Beirut, Beirut, Lebanon; ^5^ Clinical Microbiology Laboratory, Department of Pathology and Laboratory Medicine, American University of Beirut Medical Center, Beirut, Lebanon

**Keywords:** 16S rRNA, antimicrobial management, clinical specimens, diagnostic yield, clinical impact, bacterial infection, conventional culture, Lebanon

## Abstract

**Introduction:**

The identification of bacterial pathogens in the clinical setting is essential for providing optimal care and improving outcomes. The primary objective of this study was to assess the performance of the 16S test in bacterial identification from different samples and determine its impact on clinical outcomes.

**Methods:**

This was a retrospective study of patient samples collected from all age-groups at the American University of Beirut Medical Center (AUBMC), from May 2016 to December 2022. Descriptive statistics were conducted to calculate the 16S test positivity rate and to describe the different types of organisms. Univariate analyses were performed to study the clinical impact of the 16S test and its comparison to the conventional bacterial culture among different characteristics. A p ≤ 0.05 was considered statistically significant.

**Results:**

A total of 1489 specimens were submitted for the 16S test during the study period. Of the submitted tests, 395 (26%) had bacteria identified by the 16S test and/or culture. Out of the culture negative/16S positive group, the majority were from specimens collected from the skin and soft tissue system (24 out of 92, 26.1%). This was followed by musculoskeletal specimens (15 out of 92, 16.3%), and central nervous system specimens (14 out of 92, 15.2%). Pus samples had a positivity rate of 66.3% with 5 times higher odds of being positive compared to non-pus samples (25%). Overall, there were 260 identified organisms by 16S test of which the most detected organisms were *Staphylococcus* spp, *Streptococcus* spp. and *Enterobacterales.* The results revealed that 16S testing impacted management in 45.9% of the cases (83/181) showing a change in management. Antibiotic escalation was applied in 31.3% of cases (26/83). Antibiotic de-escalation occurred in 41% of cases (34/83). A change in the treating diagnosis was noted in 26.5% of cases (22/83).

**Conclusion:**

Identification of pathogens using the 16S test in combination with conventional cultures is essential in clinical diagnostics and management of infectious diseases to provide targeted therapy and improve antimicrobial stewardship. Shorter turnaround time, improved patient management, and cost-effectiveness are key factors to consider when advocating for the broader adoption of 16S testing.

## Introduction

The identification of bacterial pathogens in the clinical setting is essential for providing optimal care and improving outcomes. Until recently, traditional culture-based identification using appropriate media and Gram stains remained the standard practice. However, some bacterial pathogens are fastidious, slow growing, may not grow unless very special media are used or because they were exposed to antibiotics prior to obtaining samples for culture. Under these circumstances, the use of molecular diagnostic tools becomes advantageous. Sequencing of 16S rRNA gene (16S test) is one of these readily available tools that is becoming more utilized over the past few years. The 16S test can be applied on various clinical specimens such as tissue specimens, pleural, peritoneal, or joint effusions, cerebral spinal fluid (CSF), and blood (Ursenbach et al., 2021a). Universal primers are used to amplify bacterial nucleic acids by targeting the conserved regions; variable regions unique to each bacterial species allow the identification to the species level by PCR followed by sequencing (Cook et al., 2003). Successful identification is reported to be attainable in up to 91% of the cases (Mignard and Flandrois, 2006) in a shorter turnaround time (Mishra et al., 2019) allowing early identification, targeted therapy, favorable clinical outcomes (Fida et al., 2021) and avoiding the use of unnecessary broad antibiotics (Messacar et al., 2017).

Previous studies have demonstrated that the 16S test had both sensitivity and specificity levels above 70%. However, assessment of the impact of the 16S test on clinical outcomes and the influence on antimicrobial prescriptions are limited in the literature. Therefore, it is vital to examine the value of the 16S test on antimicrobial stewardship and cost effectiveness (Mishra et al., 2020). Due to the higher costs and technical challenges of the 16S test, it is usually reserved for patients who may benefit the most from the test to maximize diagnostic yield while taking into consideration cost effectiveness.

The primary objective of this study was to assess the performance of the 16S test in bacterial identification from different samples submitted to our laboratory and determine its impact on clinical outcomes. We specifically examined the role of the 16S test in patients where the results were discordant with conventional cultures, considering the clinical features and impact on clinical outcomes.

## Materials and methods

### Study design

This was a single center retrospective study of patient samples submitted from different clinical services at the American University of Beirut Medical Center (AUBMC) to our laboratory at the Center for Infectious Diseases Research (CIDR) located at AUBMC.

### Study population

The inclusion criteria were 1) all patients (inpatients and outpatients) with clinically suspected infection, 2) all age-groups, with 3) samples received at CIDR for 16S test collected from different body sites from May 2016 to December 2022.

### Study procedure

All 16S tests were performed at CIDR at AUBMC without prior knowledge of culture results. For patients where the culture and 16S test results were discordant (culture positive, 16S test negative or culture negative, 16S test positive or those with results showing different microbiological results), clinical data was collected. Patient electronic records were reviewed to assess whether a significant change in management or antimicrobial therapy occurred based on the 16S test result.

#### Definitions

All specimens with fluids such as joint fluid, pleural fluid, pericardial fluid and abscesses or collections were considered fluid specimens. All other specimens were considered tissue specimens including those from skin and soft tissues, muscles, heart valves, etc. In this study, gastrointestinal (GI) samples referred exclusively to specimens obtained from normally sterile sites, such as peritoneal fluid, liver abscesses, bile, and splenic tissue, collected under aseptic conditions to minimize contamination from commensal flora.

The majority of patients were suspected of having an infection. Prior antimicrobial therapy was defined as any antimicrobial therapy administered in the 2 weeks preceding sample collection.

We reviewed the impact of the 16S test on clinical management, classifying the outcomes into two main categories: ‘Change in Management’ and ‘No Change in Management.’ Among cases with a change in management (n=109), interventions included escalation or de-escalation of antimicrobial therapy, discontinuation of antibiotics, modification of the treatment diagnosis, or withholding antimicrobial therapy due to a negative 16S result. A negative 16S test refers to a result where no bacterial DNA was detected in the specimen by 16S rRNA PCR amplification and subsequent sequencing. This could be due to absence of infection, low bacterial load, or presence of bacteria not detected due to technical limitations of PCR. In cases with no change in management (n=139), factors included confirmation of culture results, severe or critical clinical status, absence of additional pathogens, coverage of identified pathogens by the current regimen, findings of contaminants, or mixed, unidentified bacterial results. We limited our analysis to 250 cases with discordant results between 16S testing and conventional culture, as these cases offered the most insight into the unique clinical contributions of 16S testing. By focusing on discordant cases—where results were either 16S-positive with culture-negative, 16S-negative with culture-positive, or both positive but with different identified organisms—we aimed to understand how 16S testing influences clinical decisions in cases where conventional methods may have limited answers or provide conflicting data. Examining key variables, such as prior antimicrobial therapy, clinical decisions made, and patient outcomes in this subset, allowed us to specifically investigate the added diagnostic and therapeutic value of 16S testing in clinical cases.

In our study, we divided the organisms detected by 16S with a negative culture into readily cultured organisms and those that require special media. In conventional microbiology, “readily cultured organisms” are those bacteria that can be easily grown using standard laboratory culture techniques and media. These organisms typically do not require special conditions or media to grow and are often identified through conventional culture methods. Conversely, “organisms that require special media to grow” are those that are more fastidious, meaning they have specific nutritional or environmental requirements that are not met by standard culture media. These organisms may require enriched or selective media, specific atmospheric conditions, or other modifications to support their growth. The challenge of culturing these organisms has led to the development of novel cultivation methods, such as the use of dilute nutrient media and simulated natural environmental conditions, to recover previously unculturable bacteria from complex bacterial communities (Vartoukian et al., 2010; Miller et al., 2018).

### Conventional microbial testing

Bacterial identification was performed using standard microbiological and biochemical techniques. Clinical specimens were first cultured on appropriate selective and differential media to allow for bacterial isolation and preliminary differentiation based on colony morphology, pigmentation, and hemolytic activity. Gram staining was subsequently conducted to classify isolates as Gram-positive or Gram-negative. Further identification was achieved through a series of biochemical assays, including catalase, coagulase, oxidase, urease, indole, and carbohydrate fermentation tests, as appropriate. These tests were interpreted according to standard bacteriological protocols to determine the species-level identity of the isolates.

#### DNA extraction and 16 rRNA PCR protocol

DNA extraction was performed using the NucleoSpin Bloodkit (Macherey-Nagel, Germany). Briefly, the specimens were incubated with lysozyme (10837059001; Sigma-Aldrich, Germany) for 20 minutes at 37°C followed by incubation with Proteinase K for 30 minutes at 70°C. The DNA was bound to the silica membrane and washed twice then eluted. Extraction kit buffers and polymerase chain reaction (PCR) reagents were routinely tested for bacterial DNA contamination. PCR was performed using the 27F/519R primers (Hassan et al., 2014).

Each PCR reaction (20 μl) consisted of 1 × HOT FIREPOL BLEND Master Mix supplemented with 7.5 mM Mgcl2 (Solis BioDyne), DNA template and nuclease free water. *Escherichia coli* chromosomal DNA was used as a positive control. The 16S PCR was performed using the C1000 Thermal Cycler (BioRad, USA). The cycling conditions were as follows: 95°C for 12 minutes corresponding to an initial denaturation, followed by denaturation over 30 cycles at 95°C for 30 seconds, annealing at 54°C for 30 seconds, elongation at 72°C for 1 minute, with a final elongation at 72°C for 5 minutes. The amplified PCR products were analyzed by gel electrophoresis through a 1% agarose gel in Tris-borate-EDTA buffer. Bands were detected by ethidium bromide staining and UV transillumination. Amplified PCR products were then purified with ExoSAP-IT PCR Product Cleanup Reagent (78201.1.ML; ThermoFisher Scientific, USA) as per the manufacturer’s instructions. Sequencing reactions were performed using the Sanger method on an ABI 3730 XL system (Applied Biosystems). Sequences were analyzed using the blast tool against the NCBI core nucleotide database with a similarity threshold of ≥90% for species-level identification.

### Statistical analysis

Statistical analysis was done using the Statistical Package for the Social Sciences (SPSS) V.28 (SPSS™ Inc., Chicago, IL USA). Descriptive statistics were conducted to identify the demographic characteristics of subjects, to calculate the 16S test positivity rate and to describe the different types of organisms. Afterward, univariate analyses using the binomial logistic regression were performed to study the clinical impact of the 16S test and its comparison to the conventional bacterial culture among different characteristics. The strength of association was interpreted using the unadjusted odds ratio (UOR) with 95% confidence interval (CI). A p ≤ 0.05 was considered statistically significant.

### Ethical considerations

This study was approved by the AUB Institutional Review Board (IRB) (Protocol number: BIO-2021-0205). The enrollment of patients occurred under complete confidentiality. The patient’s name or other identifying information was not used, each sample and its data had a specific study code. Data was collected from the patient’s chart and was coded rather than deidentified. The SPSS was secured by a username and password accessible to study team members only and was stored in password-protected computers. The data will remain and be secured for only five years after the end of the study. After that period, and after its publication, all the data will be permanently deleted.

## Results

A total of 1,489 specimens were submitted for 16S rRNA testing during the study period. Of these, 395 specimens (26%) were positive by either the 16S test and/or conventional culture. Specifically, 225 samples (15.1%) were positive by both methods, while 97 samples (6.5%) were positive by the 16S test only—92 were culture-negative (6.2%), and 5 were not cultured (0.3%) (**Supplementary Figure S1**, **Supplementary Table S1**). Among the 322 samples that were PCR-positive, 79.5% (n = 256) yielded specific bacterial identification by sequencing, including 3.7% with polymicrobial findings. Species identification was not successful in 20.5% (n = 66) of PCR-positive samples. The overall distribution of organisms detected by 16S testing and conventional culture is summarized in **Supplementary Table S2**.

Among the 1,455 specimens tested by both 16S and culture, high concordance between the two methods was observed in 1,185 cases (81.4%) (**Table 1**). Discordant results were observed in 270 cases (18.6%), with 92 (34.1%) being culture-negative but 16S-positive, and 73 (27%) being culture-positive but 16S-negative. Within the culture-negative/16S-positive group, most specimens were derived from the skin and soft tissue system (26.1%), followed by musculoskeletal (16.3%) and central nervous system (15.2%) samples (**Table 2**).

**Table 1:** Characteristics of specimens and comparison between 16S test negative and positive results.

Characteristics	All specimens	Specimens, N (%)	
		16S test negative	16S test positive
Mean age, years (± SD) * (n=257)	27.5 (± 26.2)	31.8 (± 26.3)	26.1 (± 26.0)
Comorbidities*a (n=246)	139/246 (56.5)	29/62 (46.8)	110/184 (59.8)
*Malignancy*	45/246 (18.)	8/62 (12.9)	37/184 (20.1)
*CVD*	40/246 (16.3)	13/62 (21.0)	27/184 (14.7)
*DL*	18/246 (7.3)	5/62 (8.1)	13/184 (7.1)
*DM*	27/246 (11.0)	9/62 (14.5)	18/184 (9.8)
*Pulmonary disease*	10/246 (4.1)	3/62 (4.8)	7/184 (3.8)
*Neurological disease*	12/246 (4.9)	3/62 (4.8)	9/184 (4.9)
*Other comorbidities*	54/246 (22.0)	11/62 (17.7)	43/184 (23.4)
Type of specimen (n=1489)			
*Fluid*	1161/1489 (78.0)	911/1167 (78.1)	250/322 (77.6)
*Tissue*	328/1489 (22.0)	256/1167 (21.9)	72/322 (22.4)
Bacterial culture (n=1489)			
*Negative*	1157/1489 (77.7)	1065/1167 (91.3)	92/322 (28.6)
*Positive*	298/1489 (20.0)	73/1167 (6.3)	225/322 (69.9)
*Not performed*	34/1489 (2.3)	29/1167 (2.5)	5/322 (1.6)
Culture concordance with PCR (n=1455)			
*Concordant*	1185/1455 (81.4)	1065/1138 (93.6)	120/317 (37.9)
*Discordant*	270/1455 (18.6)	73/1138 (6.4)	197/317 (62.1)
Prior antimicrobial therapy* (n=244)			
*No*	88/244 (36.1)	29/63 (46.0)	59/181 (32.6)
*Yes*	156/244 (64.7)	34/63 (54.0)	122/181 (67.4)
Management* (n=244)			
*No change in management*	135/244 (55.3)	37/63 (58.7)	98/181 (54.1)
*Change in management*	109/244 (44.7)	26/63 (41.3)	83/181 (45.9)
Change in management* (n=109)			
*ATB escalation*	26/109 (23.9)	0/26 (0.0)	26/83 (31.3)
*ATB de-escalation*	34/109 (31.2)	0/26 (0.0)	34/83 (41.0)
*Stop of ATB*	3/109 (2.8)	2/26 (7.7)	1/83 (1.2)
*Change in treating diagnosis*	22/109 (20.2)	0/26 (0.0)	22/83 (26.5)
*16S negative-ATB not initiated/not escalated/discontinued*	24/109 (22.0)	24/26 (92.3)	0/83 (0.0)
No change in management* (n=136)			
*Confirmation of culture result*	4/136 (2.9)	0/37 (0.0)	4/99(4.0)
*Severe/critical clinical status*	5/136 (3.7)	0/37 (0.0)	5/99 (5.1)
*No additional pathogens detected*	42/136 (30.9)	37/37 (100)	5/99 (5.1)
*16S pathogens covered with current ATB regimen*	25/136 (18.4)	0/37 (0.0)	25/99 (25.3)
*16S results considered contaminants*	4/136 (2.9)	0/37 (0.0)	4/99 (4.0)
*16S results revealed unidentified monomicrobial or polymicrobial infections*	56/136 (41.2)	0/37 (0.0)	56/99 (56.6)
Outcome* (n=242)			
*Recovery*	218/242 (90.1)	56/60 (93.3)	162/182 (89.0)
*Death*	8/242 (3.3)	2/60 (3.3)	6/182 (3.3)
*Other^b^*	16/242 (6.6)	2/60 (3.3)	14/182 (7.7)

*These variables were collected among discordant results only (16S test positive vs culture negative; 16S test negative vs culture positive; 16S test positive and culture positive with different identified organisms).

a There were 24 with unknown comorbidity.

b Those with other outcomes included two individuals in a vegetative state and 14 others who did not fully recover and experienced complications.

CVD, Cardiovascular Disease; DL, Dyslipidemia; DM, Diabetes Mellitus.

**Table 2:** 16S test and conventional culture results among the different specimen source systems.

System	16S negative/culture negative N (%)	16S positive/culture negative N (%)	16S negative/culture positive N (%)	16S positive/culture positive N (%)	16S positive/culture not done N (%)	16S negative/culture not done N (%)
CNS (n=447)	400 (37.6)	14 (15.2)	5 (6.8)	22 (9.8)	0 (0.0)	6 (20.7)
SST (n=291)	154 (14.5)	24 (26.1)	28 (38.4)	79 (35.1)	2 (40.0)	4 (13.8)
MSK (n=217)	144 (13.5)	15 (16.3)	17 (23.3)	40 (17.8)	0 (0.0)	1 (3.4)
Respiratory tract (n=152)	110 (10.3)	12 (13.0)	5 (6.8)	21 (9.3)	1 (20.0)	3 (10.3)
Hematology (n=121)	106 (10.0)	5 (5.4)	3 (4.1)	3 (1.3)	0 (0.0)	4 (13.8)
GI tract (n=90)	40 (3.8)	7 (7.6)	7 (9.6)	35 (15.6)	0 (0.0)	1 (3.4)
GU tract (n=43)	32 (3.0)	5 (5.4)	1 (1.4)	4 (1.8)	0 (0.0)	1 (3.4)
Cardiovascular (n=25)	19 (1.8)	3 (3.3)	0 (0.0)	3 (1.3)	0 (0.0)	0 (0.0)
Ocular (n=22)	16 (1.5)	1 (1.1)	0 (0.0)	4 (1.8)	0 (0.0)	1 (3.4)
Other sources (n=81)	44 (4.1)	6 (6.5)	7 (9.6)	14 (6.2)	2 (40.0)	8 (27.6)
**Total (n=1489)**	**1065 (71.5)**	**92 (6.2)**	**73 (4.9)**	**225 (15.1)**	**5 (0.3)**	**29 (1.9)**

CNS, Central Nervous System; SST, Skin and Soft Tissue; MSK, Musculoskeletal; GI, Gastrointestinal; GU, Genito-Urinary.

This table shows the number and proportion of 16S test and conventional culture results from each specimen source system.

The bold values in the table refer to the total number.

**Figure 1:**
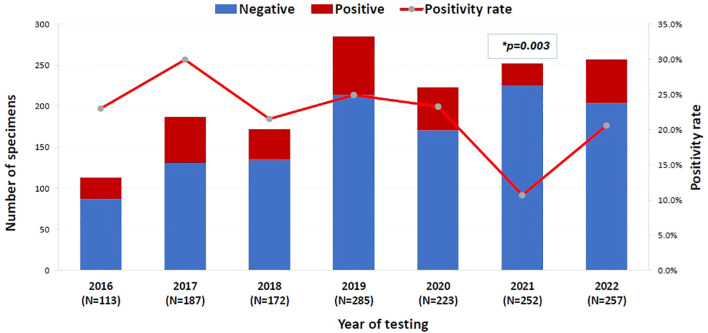
The positivity rate of 16S test across the years (N=1489). 16S rRNA PCR, 16S ribosomal Ribonucleic Acid Polymerase Chain Reaction. Binomial logistic regression analysis was used to compare the 16S rRNA PCR positivity rate across years. The year 2016 was the reference category. *P-value= 0.003, OR= 0.402, 95% CI= [0.222-0.726]. The x-axis represents the years of 16S testing from 2016 to 2022. The left y-axis is the number of 16S test negative (blue bar) and 16S test positive (red bar) specimens per year and the right y-axis is the 16S test positivity rate per year (red line).

**Figure 2:**
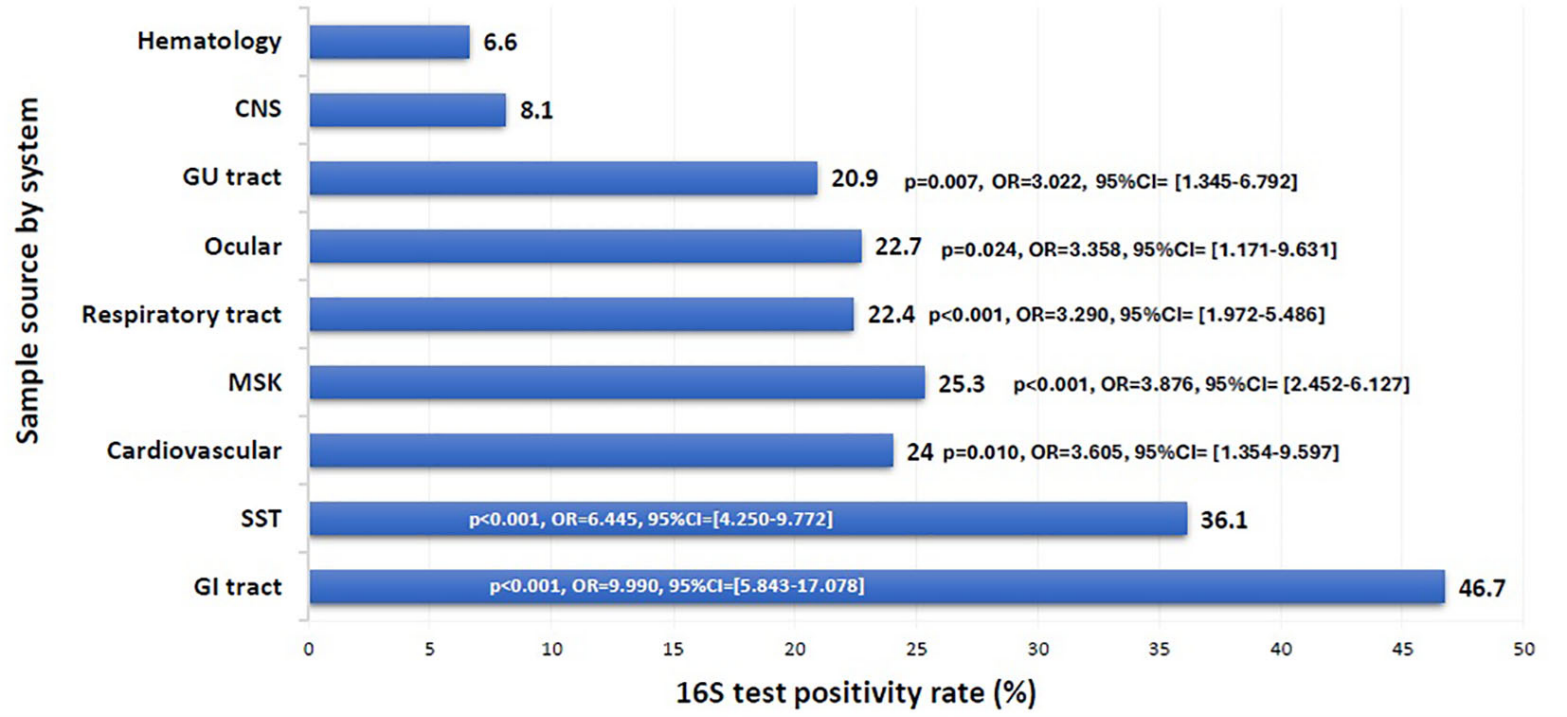
16S test positivity rate per sample source. CNS, Central Nervous System; GU, Genito-Urinary; MSK, Musculoskeletal; SST, Skin and Soft Tissue; GI, Gastrointestinal. Binomial logistic regression analysis was used to compare the 16S rRNA PCR positivity rate among sample sources by system. The CNS was the reference category. Sample sources: GI tract: peritoneal fluid, liver, spleen, abdominal wall, bile tree; SST: leg tissues, neck abscess, lymph nodes, back abscess, thigh tissues and fluids, toe swab, perianal abscess; Cardiovascular: pericardial fluid, valve, pericardial tissue, mediastinal fluid, aortic tissue, pus surrounding pacemaker; MSK: knee tissues and fluids, bone tissues, joint fluids, synovial fluids, shoulder fluids. Respiratory tract: BAL, Pleural fluid, DTA, lung tissues; Ocular: eye discharge, eye swab; GU tract: urine, urethral discharge, vaginal swab, cervical tissue; CNS: CSF, brain abscess, brain tissue; Hematology: blood, bone marrow aspirate. The x-axis represents the 16S test positivity rate. The y-axis represents the rate in each sample source system.

**Figure 3:**
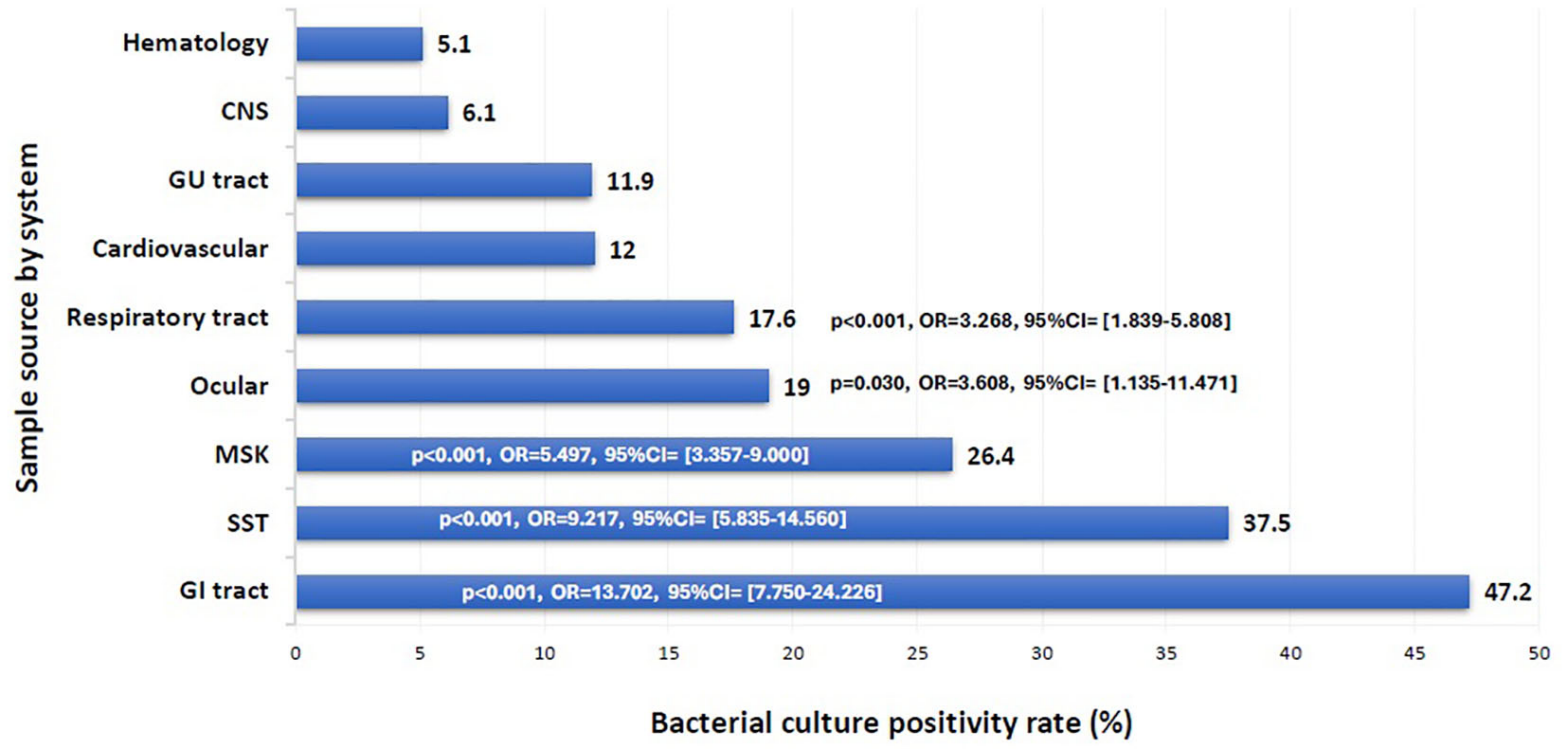
Bacterial culture positivity rate per sample source. CNS, Central Nervous System; GU, Genito-Urinary; MSK, Musculoskeletal; SST, Skin and Soft Tissue; GI, Gastrointestinal. Binomial logistic regression analysis was used to compare the bacterial culture positivity rate among sample sources by system. The CNS was the reference category. Sample sources: GI tract: peritoneal fluid, liver, spleen, abdominal wall, bile tree; SST: leg tissues, neck abscess, lymph nodes, back abscess, thigh tissues and fluids, toe swab, perianal abscess; Cardiovascular: pericardial fluid, valve, pericardial tissue, mediastinal fluid, aortic tissue, pus surrounding pacemaker; MSK: knee tissues and fluids, bone tissues, joint fluids, synovial fluids, shoulder fluids. Respiratory tract: BAL, Pleural fluid, DTA, lung tissues; Ocular: eye discharge, eye swab; GU tract: urine, urethral discharge, vaginal swab, cervical tissue; CNS: CSF, brain abscess, brain tissue; Hematology: blood, bone marrow aspirate. The x-axis represents the bacterial culture positivity rate. The y-axis represents the rate in each sample source system.

The overall positivity rate of the 16S test remained relatively stable throughout the study period, ranging from 20.6% to 29.9%, except for the year 2021, which showed a significant drop to 10.7% (p = 0.003, OR = 0.402, 95% CI: 0.222–0.726) (**Figure 1**). The likelihood of a positive 16S result was significantly higher in gastrointestinal (GI) specimens, with a positivity rate of 46.7% (42/90) (p < 0.001). GI specimens primarily included peritoneal fluid (61.8%), liver tissue (27%), splenic tissue (5.6%), abdominal wall (4.5%), and bile tree fluid (1.1%). Skin and soft tissue (SST) specimens followed, with a positivity rate of 36.1% (105/291) (p < 0.001) (**Figure 2**). Among SST specimens, 16S positivity was higher in non-surgical samples compared to surgical ones (45.9% vs. 27.6%, p = 0.005, OR = 0.449, 95% CI: 0.257–0.786) (**Table 3**). Additionally, 16S positivity in cardiovascular system specimens (24%) was significantly higher than in CNS specimens (8.1%) (p = 0.010, OR = 3.605, 95% CI: 1.354–9.597) (**Figure 3**). Similarly, culture positivity was significantly higher among GI (47.2%) and SST specimens (37.5%) compared to CNS specimens (p < 0.001).

**Table 3:** Positivity rate of the conventional culture and 16S test among the different specimen types.

Specimen types	Conventional culture					16S test				
	All tested specimens (N)	Positive (N)	Positivity rate (%)	P-value	UOR [95% CI]	All tested specimens (N)	Positive (N)	Positivity rate (%)	P-value	UOR [95% CI]
Non-pus specimens	162	49	30.2	Ref	Ref	164	41	25	Ref	Ref
Pus specimens	98	57	58.2	**<0.001**	**3.206 [1.900 - 5.409]**	98	65	66.3	**<0.001**	**5.909 [3.416 - 10.223]**
Non-surgical specimens	169	68	40.2	Ref		170	78	45.9	Ref	Ref
Surgical specimens	86	35	40.7	0.943		87	24	27.6	**0.005**	**0.449 [0.257 - 0.786]**
Fluid specimens	1136	216	19	Ref	Ref	1161	250	21.5	Ref	
Tissue specimens	319	82	25.7	**0.009**	**1.474 [1.101 - 1.973]**	328	72	22	0.871	

This table represents the number of all tested specimens, the positive specimens and the positivity rate of conventional culture and 16S test in non-pus/pus, non-surgical/surgical and tissue/fluid specimens.

The bold values in the table refer to the significant variables.

Of the 92 culture-negative/16S-positive specimens, the majority (83.7%) were fluids, including 26.1% from SST samples (**Tables 1**, **2**; **Supplementary Figure S1**). The 16S positivity rate was similar between fluid and tissue samples (21.5% vs. 22%). Notably, pus samples had a substantially higher positivity rate of 66.3%, with an approximately fivefold increased likelihood of positivity compared to non-pus samples (25%) (p < 0.001, OR = 5.909, 95% CI: 3.416–10.223) (**Table 3**). Conversely, cerebrospinal fluid (CSF) specimens showed the lowest positivity rate by 16S testing (24/414 (5.8%), p = 0.001), despite being the most frequently submitted sample type (**Table 4**).

**Table 4:** Positivity rate of the conventional culture and 16S test among the different specimen sources.

Specimen sources	Conventional culture			16S test		
	All tested specimens (N)	Positive (N)	Positivity rate (%)	All tested specimens (N)	Positive (N)	Positivity rate (%)
CSF	408	18	4.4	414	24	5.8
Tissue	250	63	25.2	254	54	21.3
Abscess	156	78	50.0	158	86	54.4
Blood	118	6	5.1	122	8	6.6
Wound	99	42	42.4	101	37	36.6
Joint fluid	86	17	19.8	87	22	25.3
Pleural fluid	67	5	7.5	69	9	13.0
Peritoneal fluid	45	21	46.7	46	20	43.5
Urine	37	2	5.4	38	7	18.4
Bone	36	7	19.4	36	6	16.7
BAL	31	8	25.8	32	10	31.3
Eye fluid	19	4	21.1	20	5	25.0
Sputum	13	6	46.2	13	8	61.5
Pericardial fluid	13	1	7.7	11	2	18.2
Cardiac valve	12	2	16.7	12	4	33.3
Other sources	65	18	27.7	74	20	27.0

This table represents the number of all tested specimens, the positive specimens and the positivity rate of conventional culture and 16S test in each specimen source.

**Figure 4:**
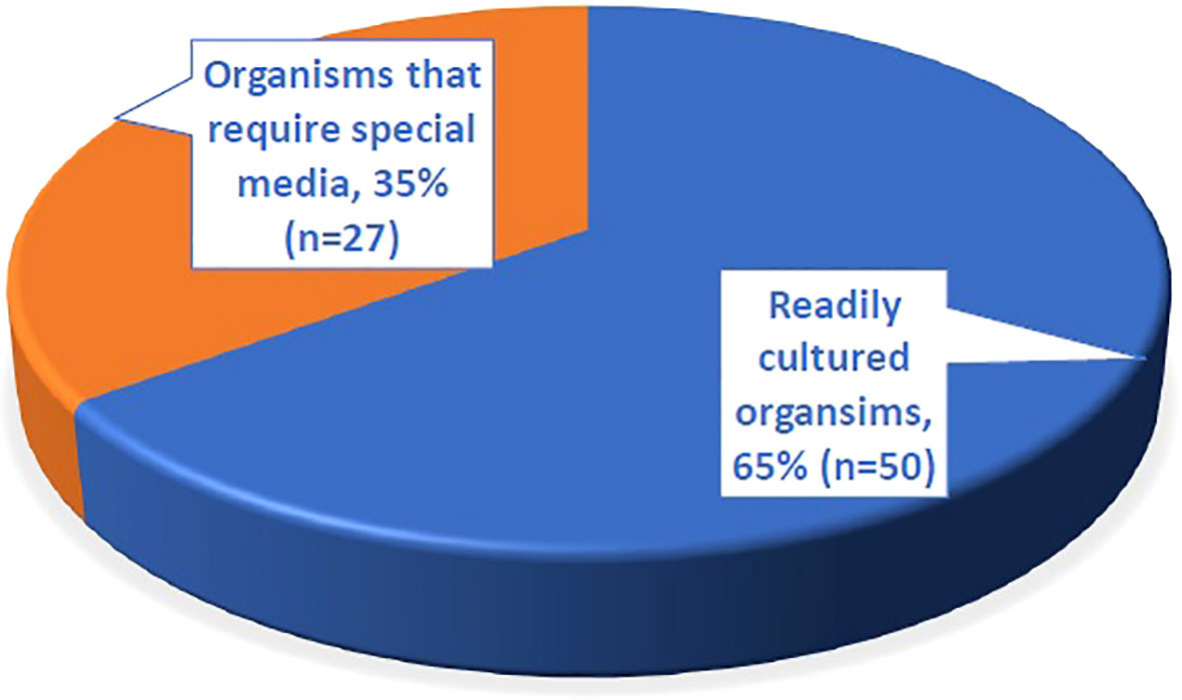
The proportion of different types of organisms identified by 16S testing from 77 culture negative samples.

A total of 260 organisms were identified through 16S sequencing (**Supplementary Figure S2**). Among the 77 organisms identified in culture-negative samples, 65% (n = 50) were organisms that are typically culturable under standard conditions, while 35% (n = 27) required special media (**Figure 4**). The commonly culturable organisms included *Streptococcus pneumoniae* (n = 9), *Streptococcus pyogenes* (n = 7), *Pseudomonas aeruginosa* (n = 5), *Escherichia coli* (n = 5), *Staphylococcus aureus* (n = 4), and *Streptococcus agalactiae* (n = 2). The group requiring special media included *Fusobacterium* spp. (n = 4), *Bacteroides fragilis* (n = 4), *Prevotella* spp. (n = 4), *Mycobacterium* spp. (n = 4), and other less common species such as *Turicella otitidis* (n = 1) and *Vibrio cholerae* (n = 2) (**Table 5**). Notably, 88.9% of the *S. pneumoniae* cases detected solely by 16S testing had prior antibiotic exposure.

**Table 5:** The overall distribution of 77^§^ organisms’ species identified by the 16S test only.

Readily cultured organisms	Organisms requiring special media
*Achromobacter xylosoxidans* (*1)	*Bacteroides fragilis* (*4)
*Enterobacter cloacae* (*1)	*Burkholderia cepacia* (*1)
*Enterococcus faecium* (*1)	*Clostridium* (*1)
*Escherichia coli* (*5)	*Corynebacterium tuberculostearicum* (*1)
*Escherichia vulneris* (*1)	*Actinomyces neuii/Staphylococcus* species (*1)
*Haemophilus influenzae* (*1)	*Francisella tularensis* (*1)
*Proteus mirabilis* (*1)	*Fusobacterium* (*1)
*Proteus vulgaris* (*2)	*Fusobacterium necrophorum* (*1)
*Pseudomonas aeruginosa* (*5)	*Fusobacterium nucleatum* (*2)
*Pseudomonas putida* (*1)	*Mycobacterium abscessus* (*3)
*Staphylococcus aureus* (*4)	*Mycobacterium marinum* (*1)
*Streptococcus agalactiae* (*2)	*Mycoplasma pneumoniae* (*1)
*Streptococcus pneumoniae* (*9)	*Prevotella bivia* (*2)
*Streptococcus pyogenes* (*7)	*Prevotella melaninogenica* (*1)
Streptococcus intermedius (*3)	*Prevotella nigrescens* (*1)
Other grouped heterogenous streptococcus (*5)	*Prevotella oris* and *Streptococcus anginosus* (*1)
	*Propionibacterium acnes* (*1)
	Tricella otitidus (*1)
	*Ureaplasma parvum* (*1)
	*Vibrio Cholerae* (*2)

^§^A total of 97 organisms resulted in 16 tests only: 77 were identified, 20 were unidentified organisms (11 with single organism and 9 mixed organisms).

Regarding unidentified organisms, some bacterial species or strains might not have closely related sequences in reference databases such as NCBI BLAST (no match). The lack of DNA purification after extraction or PCR may have generated poor-quality in the unidentified samples.

Mixed bacterial populations in a single sample can lead to ambiguous or unreadable sequences. Special software may help to identify them, but these are expensive.

The symbol “*” refers to the number of organisms.

Of the total discordant results, 64.7% (n = 156) of patients had received antibiotics within two weeks prior to sample collection. Among the culture-negative/16S-positive group, 67.4% had prior antimicrobial exposure, although this finding was not statistically significant (**Table 1**).

The clinical impact of 16S testing was observed in 45.9% of cases (83/181), resulting in changes in patient management. Among these, antibiotic escalation was implemented in 31.3% (26/83), antibiotic de-escalation in 41% (34/83), and a change in diagnosis in 26.5% (22/83). Additionally, a negative 16S result influenced the decision to withhold or discontinue antibiotic treatment in 24/109 (22%) cases. In the remaining 54.1% (98/181) of cases, no change in management was observed. This was attributed to confirmation of current treatment (4%), severe or critical patient condition necessitating continuation of therapy (5.1%), absence of additional pathogen detection (5.1%), pathogens already covered by current antibiotics (25.3%), detection of likely contaminants (4%), or presence of mixed, unidentified bacteria without specific therapeutic implications (56.6%) (**Table 1**).

**Figure 5:**
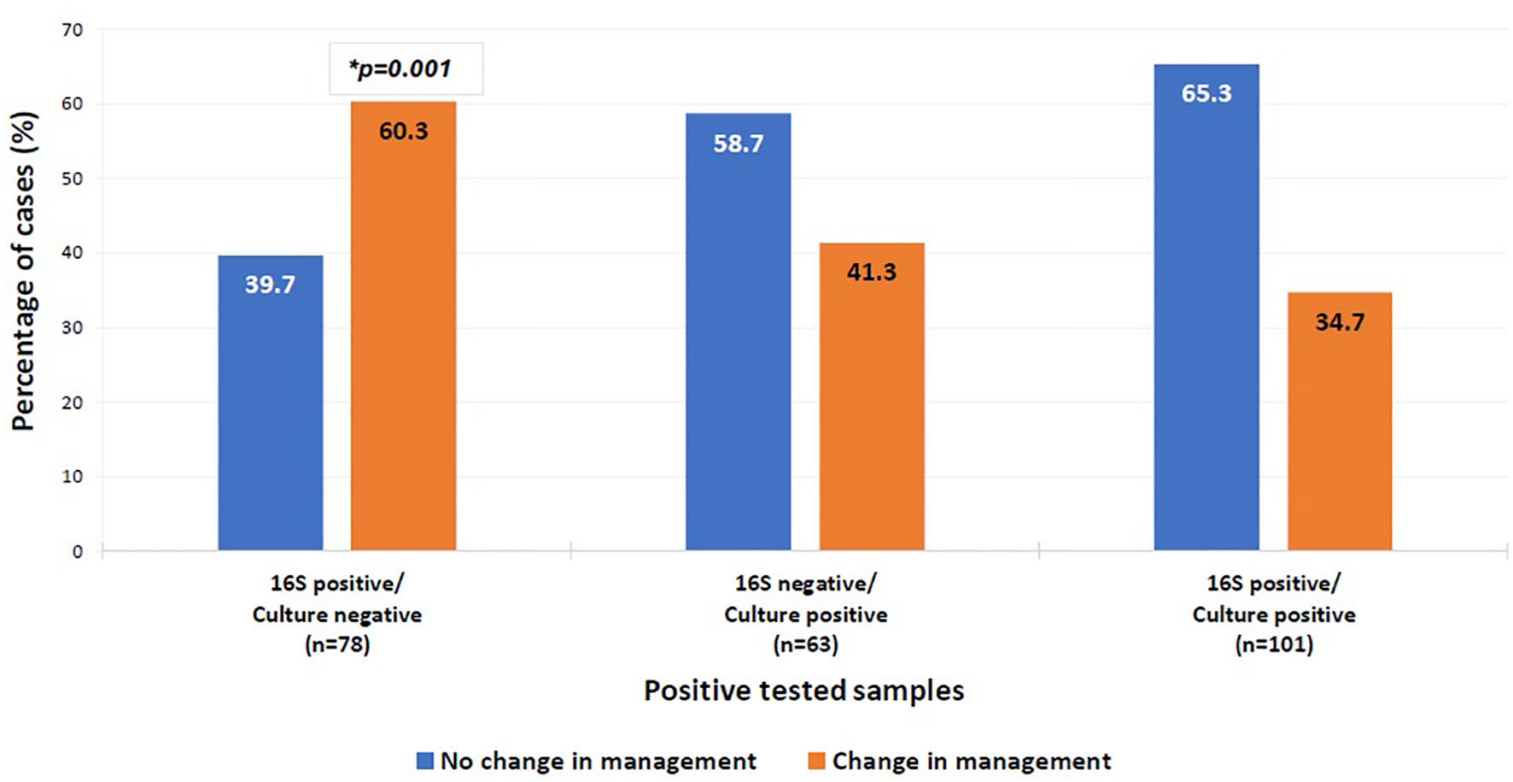
Clinical impact on management of positive tested samples. Multinomial logistic regression analysis was used to compare the clinical impact on management of the different categories of the positive tested samples. The category “16S positive/culture positive” was the reference category. *P-value=0.001, OR=2.859, 95%CI= [1.552-5.268]. The x-axis represents the positive tested samples. The y-axis represents the percentage of positive cases with a change in management (orange bar) versus the percentage of cases without a change in management (blue bar).

In cases where 16S testing was positive and no culture was requested, the clinical impact varied by sample type. Changes in management were more frequent when specimens originated from cardiac valves or urine (66.7% each), although this was not statistically significant (**Table 6**). Importantly, among culture-negative/16S-positive cases, a significant change in management was observed in 60.3% (p = 0.001, OR = 1.552–5.268) (**Figure 5**).

**Table 6:** (A) Clinical impact and (B) change in management classes among the different sources of the 16S test positive specimens.

(A)						
Source	All 16S positive specimens		Clinical impact (N)	Clinical impact (%)		P-value
CSF	12		7	58.3		0.386*
Tissue	30		12	40		
Abscess	56		28	50		
Blood	4		0	0		
Wound	20		5	25		
Joint fluid	12		7	58.3		
Pleural fluid	5		3	60		
Peritoneal fluid	6		2	33.3		
Urine	6		4	66.7		
Bone	4		1	25		
BAL	8		4	50		
Eye fluid	4		2	50		
Sputum	2		0	0		
Pericardial fluid	1		1	100		
Cardiac valve	3		2	66.7		
Other sources	8		5	62.5		
Total	181		83	45.9		

(B)						
Source	Total of 16S positive samples with clinical impact	Antibiotic escalation	Antibiotic de-escalation	Antibiotics stopped	Change in treating diagnosis	P-value
CSF	7	1	3	0	3	0.570*
Tissue	12	6	4	0	4	
Abscess	28	11	11	0	6	
Wound	5	0	1	0	4	
Joint fluid	7	3	3	0	1	
Pleural fluid	3	1	2	0	0	
Peritoneal fluid	2	1	0	0	1	
Urine	4	2	2	0	0	
Bone	1	0	1	0	0	
BAL	4	0	2	1	1	
Eye fluid	2	0	1	0	1	
Pericardial fluid	1	0	1	0	0	
Cardiac valve	2	1	0	0	1	
Other sources	5	1	3	0	1	
Total	83	26	34	1	22	

**Table 6A** represents the percentage of clinical impact for each source with positive 16S test. **Table 6B** represents the percentage of clinical impact types for each source with positive 16S test.

### Examples of clinically useful 16S test results

In the following section, several examples of clinically useful 16S test results impacting management will be presented.

In 12 patients with negative cultures likely due to prior antimicrobial use, the 16S test identified *Streptococcus pneumoniae* from various samples, such as abscesses, CSF, and pleural fluid. This allowed targeted antimicrobial therapy against *S. pneumoniae*. In several ill patients, *Bacteroides fragilis* was only detected with the 16S test leading to the addition of metronidazole to the antimicrobial regimen. On the other hand, negative 16S test results confirmed surgical bed environment in at least 6 patients prior to ventriculo-peritoneal shunt insertion. Negative 16S test results enhanced antimicrobial stewardship in cases of suspected non-infectious pleural effusions where the use of antibiotics was avoided. In the majority of cases, negative 16S test results were pivotal in determining discharge plans and the continuing need for antibiotics and their duration, positively impacting clinical management. Some additional illustrative cases follow:

1. A 57-year-old male patient with lumbar discitis was started on broad-spectrum antibiotics with repeatedly negative cultures, the 16S test detected *Vibrio cholerae* from specimens collected from disc tissue and disc fluid, leading to adjustments in the duration and management of the disease.
2. A 3-year-old patient with Truncus Arteriosus post total repair was admitted for documented endocarditis and initially treated with cefepime, vancomycin, and gentamicin. The 16S test identified *Mycoplasma pneumoniae* in mediastinal abscess fluid where blood and mediastinal fluid cultures were negative. The patient was switched to intravenous clarithromycin.
3. A 26-week premature infant was admitted to the NICU with meningitis. Despite negative CSF, blood, and urine cultures, 16S testing identified *Bacteroides fragilis* in the CSF.
4. A 36-year-old male, one month post L4-L5 discectomy, presenting with a four-day history of lower extremity weakness and pain. MRI revealed a right-sided epidural postsurgical collection. The patient was initially started on intravenous vancomycin and meropenem. However, 16S testing identified *Burkholderia* as the causative organism. This result led to a switch in therapy to intravenous ciprofloxacin, and after two weeks of marked clinical and laboratory improvement, the patient was discharged on oral ciprofloxacin.
5. *Identification of Non-Tuberculosis Mycobacterial infections*

We encountered a patient with a well-defined severe skin infection that was resistant to several antimicrobial regimens with repeated cultures showing no bacterial growth. 16S test identified *Mycobacterium abscessus* guiding the treating physician in both choice and duration of specific antimicrobial therapy. Similarly, three additional patients had *Mycobacterium abscessus* identified with the 16S test while all cultures were negative. Another patient transferred to our center for investigation and management of a pneumonia that was unresponsive to multiple broad-spectrum antibiotics with negative cultures, including bronchioalveolar lavage, the 16S test identified *Mycobacterium simiae* as the cause of pneumonia. Similarly, a 6-year-old boy with high-risk neuroblastoma, post-autologous stem cell transplant, was admitted with a suspected central venous catheter (CVC) infection. Workup showed negative blood and wound cultures, the patient was empirically started on amikacin, meropenem, and vancomycin. However, CVC swab sent for 16S testing identified *Mycobacterium abscessus* as the causative pathogen. This finding prompted a switch in therapy to intravenous cefuroxime, amikacin, and clarithromycin.

The above cases demonstrate the crucial role of 16S in enabling targeted therapy, antimicrobial de-escalation, and contribution to antimicrobial stewardship. The above illustrative cases are summarized in **Table 7**.

**Table 7:** Details of illustrative cases with 16S and culture discordant results.

Clinical diagnosis	Specimen type	16S rRNA identification	Impact on diagnosis/treatment	Antibiotic use at specimen collection	Outcome
Meningitis in a 26-week premature infant	Cerebrospinal fluid (CSF)	*Bacteroides fragilis*	Guided adjustment of antimicrobial regimen, ensuring targeted therapy	Prior antibiotics	Successful targeted treatment
Post-stem cell transplant infection (n=1) Bacteroides fragilis detection in ill patients (n=8)	Various (CSF, abscess, fluid)	*Bacteroides fragilis*	Added metronidazole, enabling tailored antimicrobial therapy	Prior antibiotics	Improved clinical outcomes through optimized treatment
Epidural abscess post-discectomy	Abscess fluid	*Burkholderia*	Allowed targeted therapy with ciprofloxacin, enabled antimicrobial de-escalation, and facilitated earlier discharge	Vancomycin, meropenem	Marked clinical improvement; discharged on oral ciprofloxacin
Endocarditis in a 3-year-old with Truncus Arteriosus	Mediastinal abscess fluid	*Mycoplasma pneumoniae*	Guided therapy switch to clarithromycin, ensuring pathogen-specific treatment	Cefepime, vancomycin, gentamicin	Successful resolution of infection
Central venous catheter infection in a 6-year-old	Catheter swab	*Mycobacteroides abscessus*	Shifted to specific therapy (cefuroxime, amikacin, clarithromycin), improving infection management	Amikacin, meropenem, vancomycin	Infection control achieved in immunocompromised pediatric patient
Patients with culture-negative infections (n=3)	Abscesses, other samples	*Mycobacterium abscessus*	Identified atypical pathogen, allowing for targeted antimicrobial management	Prior antibiotics	Improved infection control through optimized treatment
Pneumonia unresponsive to broad-spectrum antibiotics	Bronchoalveolar lavage fluid	*Mycobacterium simiae*	Identified causative pathogen, significantly altering the clinical course and optimizing management of pneumonia	Broad-spectrum antibiotics	Marked clinical improvement through tailored therapy
Central venous catheter infection in a 6-year-old	Catheter swab	*Mycobacteroides abscessus*	Therapy switched to intravenous Cefuroxime, Amikacin, and Clarithromycin, improving infection management in an immunocompromised patient	Amikacin, meropenem, vancomycin	Infection control achieved
Infected patients post-antibiotic therapy (n=9)	Various (abscess, CSF, pleural fluid)	*Streptococcus pneumoniae*	Allowed targeted antimicrobial therapy against S. pneumoniae	Prior antibiotics	Confirmed diagnosis, guided antibiotic adjustment. Targeted therapy, improved clinical management
Pleural effusion	Pleural fluid	Negative	Ruled out infectious etiology, avoided unnecessary antibiotic use	None	Enhanced antimicrobial stewardship
Pre-shunt insertion (n=6)	Surgical bed environment	Negative	Confirmed sterility, ensured safe shunt placement	None	Supported surgical decisions and reduced infection risk
Negative 16S test impact on discharge plans (n=26)	Various	Negative	Crucial in determining discharge plans, antibiotic duration, and whether to continue or discontinue antibiotics	Varies (dependent on individual case)	Positive clinical management impact, tailored antibiotic regimens and discharge planning

## Discussion

Managing patients with suspected bacterial infections can be particularly challenging in the absence of microbial identification using conventional tests. This study aimed to review the utility of combining the 16S test with traditional microbiological methods for diagnosing infections and guiding targeted antimicrobial treatment. We observed a high concordance between the 16S test and culture results. Among the 16S test-identified organisms, there were almost twice the proportion of readily cultured organisms in comparison to those that need special media. The majority of patients who had received antibiotics prior to collection of specimens had readily cultured organisms detected (71.8%) whereas their cultures were negative. This highlights the utility of the 16S test in complementing the traditional microbiology cultures in the identification of organisms in both groups. This is particularly important for “precious” specimens where sampling is obtained by invasive procedures such as biopsy, or surgery (e.g. heart valve vegetation). The impact of the 16S test is demonstrated in microbial identification in 60.3% of culture-negative samples from patients with high clinical suspicion of infection. This result is superior to a previous observation reported by Rampini et.al, where culture negative/16S positive identified pathogens formed 42.9% of the total specimens (Rampini et al., 2011).

In Lebanon, antibiotics are available over the counter without a prescription resulting in haphazard self-treatment by patients prior to evaluation by a health-care provider. This practice results in frequent negative bacterial cultures for patients who eventually present to the hospital (Reslan et al., 2022). The 16S test is particularly useful in this group of patients as positive results allow for an accurate diagnosis and tailored management. Our findings echo other studies conducted on the importance of 16S test utilization where successful de-escalation of therapy was evident after the introduction of 16S test in clinical diagnostics (O’Donnell et al., 2018). Ursenbach et al. also reported a 32% impact on antimicrobial regimen change in those with positive PCR results (Ursenbach et al., 2021a).

Overall, the 16S test was more likely to be positive with GI samples [peritoneum (61.8%), liver (27%), spleen (5.6%), abdominal wall (4.5%), and bile tree (1.1%)] in comparison to other specimen sources. This contrasts with what was previously reported in the literature with cardiovascular specimens being the samples with the most positive 16S test results (Fida et al., 2021) probably reflecting differing patient populations in these studies. Our data indicates that pus samples had the highest positivity rate for 16S testing at 66.3% (p < 0.0001), whereas CSF samples had the lowest detection rate at 5.8% (p = 0.003). Additionally, non-surgical samples had almost twice the positivity detection rate compared to surgical samples (66.3% vs. 27.6%).

This finding aligns with the literature, which consistently shows that 16S testing has a higher diagnostic yield in certain sample types. Harris and Brown reported that abscess, pus, and empyema samples had the highest diagnostic yield, with 44.9% positivity by 16S testing (Harris and Brown, 2022).

Aggarwal et al. found that pus samples were most frequently positive (34.5%; p<0.0001), while CSF samples had the lowest positivity rate (5.4%; p=0.003) (Aggarwal et al., 2020).

The low detection rate in CSF samples, despite being the most commonly referred sample type, underscores the challenges in diagnosing central nervous system infections. This is further validated by Ursenbach et al., who noted that 16S results on CSF had a significant clinical impact, particularly in patients with previous antibiotic treatment, where traditional cultures often fail (Ursenbach et al., 2021b).

The findings from our study align with and expand upon the existing literature on the impact of the 16S test on clinical management, with both similarities and notable differences in key areas such as changes in antimicrobial therapy, escalation, de-escalation, and the proportion of cases showing no impact on outcome. Our analysis showed that 45.9% of cases were impacted with a change in management as a result of 16S testing, which is notably higher compared to the 2.2% of cases impacted in Teoh et al.’s study (Teoh et al., 2021). This suggests that, in our cohort, 16S testing may have played a more significant role in guiding treatment decisions, potentially due to differences in patient demographics, infection types, or clinical practices between studies. However, it is noteworthy to mention that our analysis was solely done on discordant results which would affect the findings in comparison to other studies. Despite being limited to 45.9% of cases, the impact of 16S testing on clinical management remains substantial. In clinical practice, a diagnostic tool that leads to a change in management for nearly half of patients is valuable. While 16S testing did not significantly influence cases concordant with culture results, it still contributed to improved clinical outcomes by enabling faster pathogen identification and more targeted treatments. This may reduce reliance on broad-spectrum antibiotics, lowering the risk of antimicrobial resistance while enhancing infection treatment with more appropriate antimicrobial agents. The high rate of management change observed in our findings aligns with Ursenbach et al., who reported a substantial effect, with 50% of cases showing a change in antimicrobial regimen (Ursenbach et al., 2021a).

When exploring management changes resulting from 16S test results in our study, escalation of antibiotics occurred in 31.3% of cases where a change in management occurred, closely matching the 39% escalation rate in Ursenbach et al.’s findings (Ursenbach et al., 2021b). This consistency suggests that in specific cases, 16S testing reliably supports the decision to intensify therapy based on identified pathogens, underscoring its role in addressing previously unidentified or unexpected infections. On the other hand, de-escalation of antibiotics was observed at a higher rate than the 15.6% in Akram et al. and the 21% noted by O’Donnell et al. (Akram et al., 2017). This discrepancy may reflect differences in clinical thresholds for reducing therapy intensity or the types of pathogens commonly encountered in our study compared to others. The high de-escalation rate in the current study indicates that 16S testing provided sufficient information to support more targeted therapy and reduce unnecessary antibiotic exposure, aligning with antimicrobial stewardship goals.

Our study also captured the impact of 16S testing in situations where antibiotics were discontinued or where a change in the diagnosis prompted treatment adjustments. Ursenbach et al. similarly reported significant rationalization of antibiotic treatment in 50% of cases, suggesting that the 16S test consistently enables tailored adjustments in diverse clinical settings (Ursenbach et al., 2021b).

Lastly, our study revealed that 56% of cases had no change in management, compared to the majority of cases in Teoh et al.’s findings (Teoh et al., 2021). This outcome indicates that while the 16S test frequently supports antimicrobial decisions, a substantial proportion of cases still benefit from confirmation of adequate management without requiring alterations. In our cohort, factors such as confirmation of culture results, severe clinical status, or contaminants identified by 16S testing contributed to this lack of change, reinforcing its utility as a complementary diagnostic tool.

In this study, the 16S sequencing success rate in PCR-positive cases was 79.5%. In the remaining cases (20.5%), species-level identification may have been hindered by the presence of mixed bacterial populations. The latter may contribute to the generation of ambiguous or unreadable sequences. Additionally, some bacterial species or strains might not have closely related sequences in reference databases such as NCBI BLAST. Furthermore, lower DNA purity in some unidentified samples may have generated poor quality. Similar to our findings, a study by Fida et al. reported that 22% of samples with positive 16S PCR were not identified by Sanger sequencing (Fida et al., 2021). Another study demonstrated that 2.8% of samples with a positive 16s amplification product indicated suspected mixed infections (Eamsakulrat et al., 2022). The authors attributed the failure of species identification in additional 12.3% of specimens to unsuccessful Sanger sequencing.

Furthermore, negative 16S test results led to no initiation, escalation, or discontinuation of antibiotics in a quarter of the cases reported in the current study. This finding aligns with the literature, which supports the utility of negative 16S results in guiding clinical decisions, particularly in antimicrobial stewardship. Gilbert et al. demonstrated that negative 16S results were associated with shorter lengths of antibiotic therapy and higher rates of antibiotic discontinuation in culture-negative patients (Gilbert et al., 2017). Similarly, O’Donnell et al. found that negative 16S results contributed to the discontinuation of antimicrobials in 8% of cases, highlighting its role in preventing unnecessary antibiotic use (O’Donnell et al., 2018). These studies underscore the importance of negative 16S results in clinical decision-making, particularly in avoiding unnecessary antimicrobial therapy, which is crucial for antimicrobial stewardship (Gilbert et al., 2017; O’Donnell et al., 2018).

The samples that were negative by both 16S and culture were not included in the discordant analysis but were part of the total cohort to provide a comprehensive overview of test performance. The double negatives are important in measuring the specificity of the 16S test. This group includes patients with no bacterial infection, cases with prior effective antibiotic treatment, and non-bacterial etiologies such as viral, autoimmune, or sterile inflammatory conditions. Importantly, in some cases, these double-negative results served a crucial confirmatory role, for example, confirming the sterility of the surgical field before ventriculoperitoneal shunt placement or other invasive procedures. Such findings provided reassurance to clinicians and directly influenced management decisions by supporting the safety of proceeding without antimicrobial coverage or delaying interventions. However, careful clinical correlation would be essential in interpreting such negative microbiological findings, as molecular diagnostics alone cannot exclude infection in the appropriate context.

### Limitations

One of the limitations of this study is its retrospective nature, where some of the cases had missing data due to incomplete notes. Importantly, the decision-making process is not always documented clearly in medical records, possibly leading to under or overestimation of the impact of 16S test results on clinical management. Moreover, due to the heterogeneous sources of samples submitted, comparisons regarding sensitivity of the test and its impact on outcome according to sample source were not possible. Including cost-effectiveness as an essential factor associated with the routine use of 16S testing is warranted in future studies. Additionally, sequencing analysis could have been improved to enhance species identification in polymicrobial infections. A key limitation of our approach was the inability to resolve polymicrobial infections using Sanger sequencing. In cases where multiple bacterial templates were present, the resulting chromatograms frequently produced overlapping signals, rendering species-level identification inconclusive. These findings were reported as mixed bacterial populations and, in the absence of definitive identification, did not influence clinical decision-making. The integration of advanced sequencing technologies such as next-generation sequencing (NGS) or Whole Genome Sequencing (WGS), along with specialized bioinformatics tools, may facilitate more accurate characterization of polymicrobial samples and enhance the clinical applicability of 16S testing in future investigations.

While this case of lumbar discitis associated with *Vibrio cholerae* is unusual, and the patient’s clinical response supported its role as the causative pathogen, we cannot exclude the possibility of misidentification or sample contamination as an alternative explanation, highlighting the need for careful interpretation of molecular findings in rare presentations.

## Conclusion

This study represents the largest scale study conducted in low- and middle-income countries (LMIC), summarizing findings from 16S rRNA-targeted sequencing conducted on samples suspected of clinical infection. This is especially important because many LMIC have high rates of multi-drug-resistant bacterial infections and lack appropriate antimicrobial stewardship (Khafaja et al., 2024; Zakhour et al., 2025). Identification of pathogens using the 16S test in combination with conventional cultures is essential in clinical diagnostics and management of infectious diseases, particularly those already on antibiotics at the time of sampling to provide targeted therapy and improve antimicrobial stewardship. Shorter turnaround time, improved patient management, and cost-effectiveness are key factors to consider when advocating for the broader adoption of 16S testing. This study provides support for the wider implementation of this test. Further studies are needed on a larger scale to confirm our findings and cover unaddressed factors.

## Data Availability

The datasets presented in this article are not readily available due to privacy and ethical restrictions. Requests to access the datasets should be directed to the corresponding author(s).
