# Supplementary material for: The clinical impact of 16S ribosomal RNA PCR and sequencing in the identification of bacterial infections: a 7-year report from a Lebanese tertiary care center
Source: Front Cell Infect Microbiol. 2025 Aug 7;15:1619640. doi: 10.3389/fcimb.2025.1619640 (PMC12367777; doi:10.3389/fcimb.2025.1619640)
Supplement: Supplementary file 1 [file Table1.docx]

**Figure 1S. Outcome of the 16S test versus culture for identifying bacteria in various sample sources**

**Table 1S. List of identified organisms by fluid and tissue samples according to the different positive tests**

| **16S test positive - Culture positive** | | **16S test positive - Culture negative** | | **16S test positive - Culture not done** | | **16S test negative - Culture positive** | |
| --- | --- | --- | --- | --- | --- | --- | --- |
| ***Fluid*** | ***Tissue*** | ***Fluid*** | ***Tissue*** | ***Fluid*** | ***Tissue*** | ***Fluid*** | ***Tissue*** |
| *Staphylococcus aureus (N=28)* | *Staphylococcus aureus (N=8)* | *Streptococcus pneumoniae (N=9)* | *Staphylococcus aureus (N=2)* | *Proteus mirabilis* | *Mycobacterium marinum* | *Staphylococcus aureus (N=7)* | *Staphylococcus aureus (N=5)* |
| *Escherichia coli (N=21)* | *Staphylococcus epidermidis/coagulase negative (N=6)* | *Streptococcus pyogenes (N=5)* | *Prevotella* species (N=2) | *Streptococcus intermedius* |  | *Staphylococcus epidermidis/coagulase negative (N=6)* | *Staphylococcus epidermidis/coagulase negative (N=5)* |
| *Pseudomonas aeruginosa (N=13)* | *Escherichia coli (N=4)* | *Bacteroides fragilis (N=4)* | *Enterobacter* species (N=2) | *Escherichia vulneris* |  | Other *staphylococcus* species (N=3) | *Pseudomonas aeruginosa (N=4)* |
| *Klebsiella pneumoniae (N=12)* | *Klebsiella oxytoca (N=4)* | *Escherichia coli (N=4)* | *Escherichia coli* | Mixed unidentified bacteria |  | *Pseudomonas aeruginosa (N=2)* | *Pseudomonas stutzeri* |
| *Staphylococcus epidermidis (N=11)* | *Burkholderia cepacia (N=4)* | Other grouped *streptococcus* heterogenous group *(N=4)* | *Pseudomonas aeruginosa* |  |  | *Streptococcus constellatus (N=2)* | *Streptococcus pyogenes* |
| *Streptococcus pyogenes (N=7)* | *Pseudomonas aeruginosa (N=4)* | *Fusobacterium* species (N=4) | *Streptococcus pyogenes* |  |  | *Stenotrophomans maltophilia (N=2)* | *Kocuria rhizophila* |
| *Streptococcus constellatus/intermedius (N=6)* | *Streptococcus intermedius (N=4)* | Pseudomonas aeruginosa (N=3) | *Vibrio cholerae* |  |  | *Escherichia coli (N=2)* | *Burkholderia cepacia* |
| *Fusobacterium* species (N=6) | *Streptococcus pyogenes (N=3)* | Other *staphylococcus* species (N=3) |  |  |  | *Salmonella enteritidis (N=2)* | *Microbacterium paraoxydans* |
| *Enterococcus* species (N=6) | *Klebsiella pneumoniae (N=2)* | *Mycobacterium abscessus (N=3)* |  |  |  | *Streptococcus viridans* | *Micrococcus* species |
| *Enterobacter* species (N=6) | *Bacteroides* species (N=2) | *Streptococcus agalactiae (N=2)* |  |  |  | *Streptococcus pneumoniae* | Other mixed bacteria**** (N=4) |
| *Streptococcus viridans (N=5)* | *Fusobacterium nucleatum* | *Streptococcus intermedius (N=2)* |  |  |  | *Weissella confusa* |  |
| Other grouped *streptococcus* heterogenous group *(N=5)* | *Acinetobacter baumannii* | *Staphylococcus aureus (N=2)* |  |  |  | *Enterobacter cloacae* |  |
| Other *staphylococcus* species (N=5) | *Mycobacterium marinum* | *Proteus vulgaris (N=2)* |  |  |  | *Moraxella* |  |
| Other *pseudomonas* species (N=5) | *Morganella morganii* | *Prevotella* species (N=2) |  |  |  | *Haemophilus parainfluenzae* |  |
| *Bacteroides* species (N=4) | *Stenotrophomonas maltophilia* | *Burkholderia cepacia* |  |  |  | *Ochrobactrum anthropi* |  |
| *Streptococcus pneumoniae (N=3)* | *Achromobacter xylosoxidans* | *Haemophilus influenzae* |  |  |  | *Dermabacter hominis* |  |
| *Prevotella* species (N=3) | *Enterobacter cloacae* | *Propionibacterium acnes* |  |  |  | *Micrococcus luteus* |  |
| *Burkholderia cepacia (N=2)* | *Staphylococcus haemolyticus* | *Francisella tularensis* |  |  |  | *Mycobacterium tuberculosis* |  |
| *Salmonella enteritidis (N=2)* | *Salmonella enteritidis* | *Mycoplasma pneumoniae* |  |  |  | *Achromobacter xylosoxidans* |  |
| *Stenotrophomonas maltophilia (N=2)* | *Streptococcus viridans* | *Achromobacter xylosoxidans* |  |  |  | Other mixed bacteria*** (N=5) |  |
| *Acinetobacter* species (N=2) | *Streptococcus mitis* | *Enterococcus faecium* |  |  |  |  |  |
| *Mycobacterium* species (N=2) | *Pseudomonas stutzeri* | *Vibrio cholerae* |  |  |  |  |  |
| *Klebsiella aerogenes* | *Lactobacillus* species | *Corynebacterium tuberculostearicum* |  |  |  |  |  |
| *Haemophilus aphrophilus* | Other mixed bacteria** (N=12) | *Clostridium* | |  |  |  |  |
| *Brevibacterium cusei* |  | *Ureaplasma parvum* |  |  |  |  |  |
| *Citrobacter freundii* |  | *Pseudomonas putida* |  |  |  |  |  |
| *Corynebacterium striatum* |  | *Actinomyces neuii & Staphylococcus* species |  |  |  |  |  |
| *Proteus mirabilis* |  |  | |  |  |  |  |
| *Porphyromonas somerae* |  |  |  |  |  |  |  |
| *Morganella Morganii* |  |  |  |  |  |  |  |
| *Serratia marcescens* |  |  |  |  |  |  |  |
| *Achromobacter xylosoxidans* |  |  |  |  |  |  |  |
| *Eikenella corrodens* |  |  |  |  |  |  |  |
| *Moraxella catarrhalis* |  |  |  |  |  |  |  |
| *Ochrobactrum anthropi* |  |  |  |  |  |  |  |
| *Parabacteroides distasonis* |  |  |  |  |  |  |  |
| *Clostridium hiranonis* |  |  |  |  |  |  |  |
| *Lactobacillus iners* |  |  |  |  |  |  |  |
| Other mixed bacteria* (N=11) |  |  |  |  |  |  |  |

******Streptococcus group F & Haemophilus parainfluenzae; Streptococcus parasanguinis & Enterococcus; Staphylococcus aureus & Streptococcus viridans; Acinetobacter & Stenotrophomonas maltophilia; Enterobacter cloacae & Klebsiella pneumoniae; Escherichia coli & Fusobacterium species; Escherichia coli & Salmonella enterica; Escherichia coli & Streptococcus intermedius; Escherichia coli & Streptococcus viridans; Klebsiella pneumoniae & Escherichia coli & Enterococcus species; Acinetobacter lwoffi & Streptococcus pneumoniae.*

***Escherichia coli & Klebsiella pneumoniae; Escherichia coli & Streptococcus anginosus; Streptococcus anginosus & Prevotella oris; Pseudomonas aeruginosa & Achromobacter xylosoxidans; Pseudomonas aeruginosa & Enterococcus faecium; Serratia marcescens & Acinetobacter baumannii; Staphylococcus aureus & Streptococcus pyogenes; Staphylococcus epidermidis & Stenotrophomonas maltophilia; Staphylococcus epidermidis & Bacteroides fragilis; Streptococcus agalactiae & Escherichia coli; Enterobacter aerogenes & Stenotrophomonas maltophilia; Streptococcus anginosus & Klebsiella pneumoniae.*

****Enterococcus species & Stenotrophomonas maltophilia; Proteus mirabilis & Pseudomonas aeruginosa; Staphylococcus epidermidis & Staphylococcus haemolyticus; Lactobacillus rhamnosus & Actinomyces odontolyticus; Proteus mirabilis & Escherichia coli & Klebsiella pneumoniae.*

*****Klebsiella oxytoca & Enterococcus faecalis; Pseudomonas aeruginosa & Enterococcus faecium; Pseudomonas aeruginosa & Ochrobactrum intermedium; Staphylococcus aureus & Streptococcus agalactiae.*

**Table 2S. Overall distribution of the identified organisms by conventional culture and 16S test.**

|  | Culture | 16S test |
| --- | --- | --- |
| *Staphylococcus spp.* | 90 | 46 |
| *Streptococcus spp.* | 36 | 53 |
| *Escherichia coli* | 30 | 24 |
| *Pseudomonas spp.* | 29 | 27 |
| *Klebsiella spp.* | 15 | 14 |
| *Fusobacterium spp.* | 0 | 13 |
| *Bacteroides spp.* | 0 | 11 |
| *Enterobacter spp.* | 7 | 6 |
| *Prevotella spp.* | 1 | 8 |
| *Mycobacterium spp.* | 2 | 7 |
| *Burkholderia cepacia* | 5 | 4 |
| *Stenotrophomonas maltophilia* | 6 | 2 |
| *Enterococcus spp.* | 5 | 3 |
| *Proteus spp.* | 1 | 4 |
| *Salmonella enteritidis* | 5 | 0 |
| *Achromobacter spp.* | 2 | 3 |
| *Acinetobacter spp.* | 3 | 2 |
| *Morganella morganii* | 2 | 2 |
| *Clostridium spp.* | 0 | 2 |
| *Eikenella corrodens* | 2 | 0 |
| *Micrococcus spp.* | 2 | 0 |
| *Ochrobactrum anthropi* | 2 | 0 |
| *Ureaplasma spp.* | 0 | 2 |
| *Vibrio cholerae* | 0 | 2 |
| *Corynebacterium spp.* | 1 | 1 |
| *Haemophilus spp.* | 1 | 1 |
| *Lactobacillus spp.* | 1 | 1 |
| *Serratia marcescens* | 1 | 1 |
| *Brevibacterium* ***casei*** | 1 | 0 |
| *Dermabacter hominis* | 1 | 0 |
| *Kocuria rhizophila* | 1 | 0 |
| *Microbacterium paraoxydans* | 1 | 0 |
| *Moraxella* | 1 | 0 |
| *Weissella confusa* | 1 | 0 |
| *Escherichia vulneris* | 0 | 1 |
| *Francisella tularensis* | 0 | 1 |
| *Mycoplasma pneumoniae* | 0 | 1 |
| *Neisseria subflava* | 0 | 1 |
| *Parabacteroides distasonis* | 0 | 1 |
| *Porphyromonas somerae* | 0 | 1 |
| *Propionibacterium acnes (Cutibacterium acnes)* | 0 | 1 |
| *Turicella otitidis* | 0 | 1 |
| *Mixed bacterial infections* | 40 | 9 |
| *Unidentified bacteria* | 3 | 28* |
| *Unidentified mixed bacteria* | 0 | 38** |
| *Total* | **298** | **322** |

*Some bacterial species or strains might not have closely related sequences in reference databases such as NCBI BLAST (no match). 
The lack of DNA purification after extraction or PCR may have generated poor-quality or degraded DNA in the unidentified samples.

**Mixed bacterial populations in a single sample can lead to ambiguous or unreadable sequences. Special software may help to identify them, but these are expensive.

**(A)**

**(B)**

*Streptococcus viridans include *Streptococcus intermedius* (*11), *Streptococcus mitis* (*5); *Streptococcus anginosus/Prevotella oris* (*1); *Streptococcus gallolyticus* (*1); *Streptococcus oralis* (*1); *Streptococcus thermophilus* (*1); *Streptococcus viridans* (*1).

**(C)**

**Figure 2S. The number of 16S positive samples among the most commonly detected organisms categorized into (A*) Staphylococcus* spp., (B) *Streptococcus* spp., and (C) *Enterobacterales*.**
